# Management of Circumferential Cervical Benign Symmetric Lipomatosis: A Case Report of Staged Surgical Resection

**DOI:** 10.7759/cureus.87074

**Published:** 2025-06-30

**Authors:** Ryo Fukuda, Hirotoshi Ohara, Yusuke Shimizu, Taiki Nagatsuka, Edward H Ntege

**Affiliations:** 1 Plastic and Reconstructive Surgery, University of the Ryukyus Hospital, Ginowan, JPN; 2 Plastic and Reconstructive Surgery, International University of Health and Welfare, Mita Hospital, Tokyo, JPN

**Keywords:** airway management, benign symmetric lipomatosis, case report, madelung disease, neck surgery, staged excision, submental laxity

## Abstract

Benign symmetric lipomatosis (BSL), or Madelung disease, is a rare condition characterized by non-encapsulated, symmetrical adipose tissue accumulation. Although uncommon, circumferential cervical involvement can pose a life-threatening risk of airway obstruction. We report a 58-year-old Japanese man with chronic alcohol dependence presenting with progressive bilateral neck swelling, dysphagia, and nocturnal respiratory distress. Initial magnetic resonance imaging revealed 21 mm of adipose tissue at the C4/5 level. Following posterior cervical lipectomy and subsequent disease recurrence, imaging five years later demonstrated progression to 70 mm of circumferential adipose proliferation with impending airway compromise. A quadrant-based, four-stage open resection protocol was performed between March 2023 and February 2024, utilizing sequential lateral, anterior, and posterior approaches. This staged approach successfully avoided tracheostomy while removing approximately 2,100 grams of adipose tissue. At 16-month follow-up, cervical thickness reduced to 48 mm with complete resolution of dysphagia and respiratory symptoms. Residual submental laxity from platysmal diastasis and submandibular gland hypertrophy remained, though secondary procedures were deferred due to questionable alcohol abstinence evidenced by persistently elevated γ-GTP levels. This case demonstrates that systematic staged resection can safely manage circumferential cervical BSL while preserving airway integrity. However, sustained alcohol abstinence remains critical for preventing recurrence, and comprehensive functional assessments should guide future management protocols for this rare condition.

## Introduction

Benign symmetric lipomatosis (BSL), also known as Madelung disease or Launois-Bensaude syndrome, is a rare disorder characterized by progressive, non-encapsulated, symmetrical adipose tissue proliferation with a predilection for the cervicothoracic region [1-3]. Although Madelung disease was first documented in 1846 and comprehensively described several decades later, its diagnosis and treatment remain challenging [3-5].

BSL demonstrates a marked male predominance with a ratio of 15:1 to 30:1, typically affecting individuals aged 30 to 60 years [6]. Geographic distribution suggests increased prevalence among Mediterranean populations, with an estimated incidence of approximately 1 in 25,000 [7,8]. The disorder requires careful differentiation from simple obesity and multiple lipomatosis syndromes through combined clinical and radiological evaluation.

The pathophysiology remains incompletely understood, though chronic alcohol consumption, documented in up to 90 percent of cases, appears central through suppression of β-adrenergic lipolytic pathways and induction of mitochondrial dysfunction [8,9]. Genetic investigations have identified familial cases associated with mutations in mitofusin 2 (MFN2) and hormone-sensitive lipase (LIPE) genes, as well as mitochondrial DNA variants linked to myoclonic epilepsy with ragged red fibers syndrome (MERRF syndrome) [9-11].

Anatomically, the Enzi classification defines two primary phenotypes: type I characterized by cervicothoracic adipose infiltration creating the classic "Madelung collar," and type II with pseudo-athletic truncal fat distribution [12]. Circumferential cervical involvement, where adipose deposits completely encircle vital structures, represents a particularly hazardous variant. This pattern predisposes patients to progressive dysphagia, obstructive sleep apnea, and critical perioperative airway management challenges [13,14].

Therapeutic management remains predominantly surgical for symptomatic or cosmetically disfiguring disease. Open lipectomy enables en bloc excision with effective hemostasis but requires extensive incisions. Alternative techniques, including power-assisted and ultrasound-assisted liposuction, offer less invasive approaches but demonstrate recurrence rates of 30 to 60 percent due to incomplete excision of fibrous adipose tissue [15,16]. The medical literature regarding optimal surgical protocols for circumferential cervical BSL consists primarily of isolated case reports, with no evidence-based consensus on staging approaches or management of postoperative deformities.

We present a 58-year-old man with extensive circumferential cervical BSL causing severe functional impairment, successfully managed using a novel quadrant-based, four-stage open resection protocol. Our surgical methodology, quantitative outcomes, and comprehensive analysis aim to establish a reproducible framework for managing this rare but potentially life-threatening condition.

## Case presentation

A 58-year-old Japanese man presented to the Department of Plastic Surgery, International University of Health and Welfare, Mita Hospital, Tokyo, Japan, with a history of progressively enlarging bilateral posterior cervical masses accompanied by worsening nocturnal respiratory distress. He denied pain, fever, or unexplained weight loss.

His medical history included documented chronic liver dysfunction with transaminitis and mixed dyslipidemia confirmed by laboratory findings, as well as atrial fibrillation, though specific medication details were not available in the medical records. He reported chronic heavy alcohol consumption - approximately 900 mL of shōchū daily (25% alcohol by volume), equivalent to ~180 g of ethanol per day over several decades. He had no known allergies or family history suggestive of similar conditions.

On initial clinical assessment, palpation revealed soft, symmetrical, non-tender subcutaneous masses in the posterior cervical and upper back regions, with relatively clear boundaries from the surrounding subcutaneous tissue. The overlying skin appeared normal without discoloration or telangiectasia.

Laboratory investigations revealed a significant elevation of liver enzymes with preserved glycemic control. Dyslipidemia was medically managed, and no additional metabolic abnormalities of diagnostic significance were observed (Table 1). The marked elevation of hepatic transaminases (AST 85 U/L, ALT 36 U/L) and γ-GTP (188 U/L) was consistent with alcohol-related hepatocellular injury.

**Table 1:** Laboratory findings at initial presentation, preoperative assessment, and follow-up ALP: alkaline phosphatase; ALT: alanine aminotransferase; AST: aspartate aminotransferase; BUN: blood urea nitrogen; Cl: chloride; CRP: C-reactive protein; eGFR: estimated glomerular filtration rate; GOT: glutamic-oxaloacetic transaminase; GPT: glutamic-pyruvic transaminase; γ-GTP: gamma-glutamyl transpeptidase; HbA1c: hemoglobin A1c; HDL: high-density lipoprotein; K: potassium; LDL: low-density lipoprotein; Na: sodium; RBC: red blood cell; WBC: white blood cell Notes: (-) indicates test not performed on that date; Follow-up values ​​show the most recent data.

Parameter	Initial Visit (2016/09/08)	Preoperative Day (2022/07/26)	Postoperative Days (2025/05/13)	Reference Range	Units
Liver Function Tests					
AST (GOT)	85	77	76	13-30	U/L
ALT (GPT)	36	17	29	10-42	U/L
ALP	249	-	70	106-322	U/L
γ-GTP	188	274	134	13-64	U/L
Total Bilirubin	1.4	0.9	1.1	0.4-1.5	mg/dL
Lipid Profile					
Total Cholesterol	167	154	179	142-248	mg/dL
LDL Cholesterol	86	-	-	65-163	mg/dL
HDL Cholesterol	70	-	-	40-90	mg/dL
Triglycerides	56	114	91	40-234	mg/dL
Glucose Metabolism					
Fasting Glucose	96	252	109	73-109	mg/dL
HbA1c	5.3	-	5.3	4.9-6.0	%
Renal Function					
BUN	12.9	7.5	8.4	8-20	mg/dL
Creatinine	0.74	0.71	0.71	0.65-1.07	mg/dL
eGFR	84.1	85.5	84.4	>90	mL/min/1.73m²
Electrolytes					
Sodium (Na)	142	141	142	138-145	mEq/L
Potassium (K)	4.6	3.8	4.2	3.6-4.8	mEq/L
Chloride (Cl)	103	103	104	101-108	mEq/L
Other Parameters					
Total Protein	6.8	6.1	6.7	6.6-8.1	g/dL

**Figure 1:**
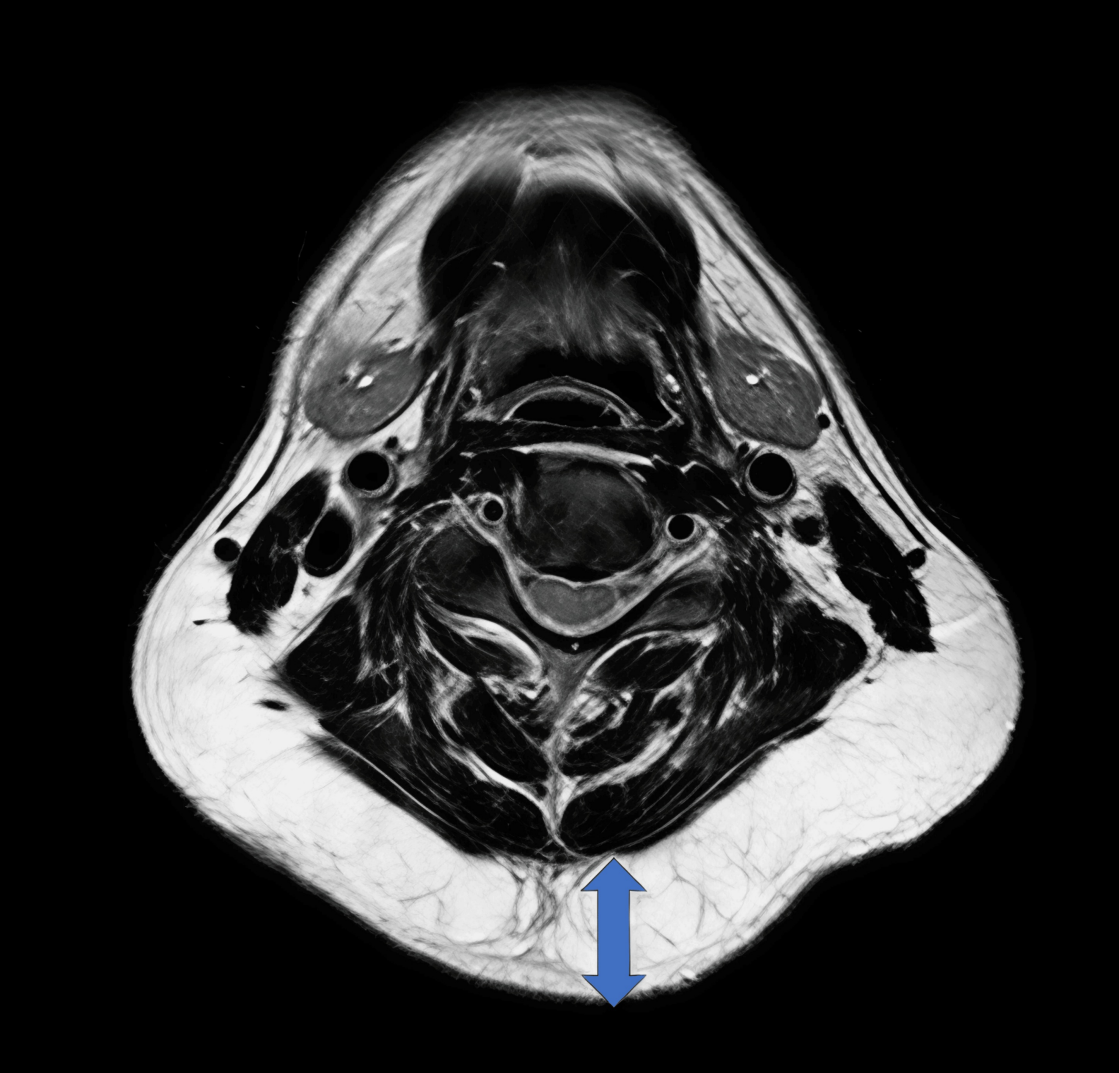
Initial MRI imaging of cervical benign symmetric lipomatosis T1-weighted axial image showing symmetrical, non-encapsulated adipose proliferation measuring 21 mm at the C4/5 vertebral level, with predominance in the posterior cervical region.

Initial magnetic resonance imaging (MRI) revealed symmetrical, non-encapsulated adipose proliferation demonstrating high signal intensity on both T1-weighted and T2-weighted sequences, measuring approximately 21 mm in maximum anteroposterior diameter at the C4/5 vertebral level (Figure 1).

The differential diagnoses included generalized obesity, multiple encapsulated lipomas, familial multiple lipomatosis, liposarcoma, Cushing syndrome, angiolipoma, and drug-induced lipomatosis. Given the symmetric distribution, lack of encapsulation, benign imaging characteristics, and clinical presentation in the setting of chronic alcoholism, the diagnosis of BSL was established.

**Figure 2:**
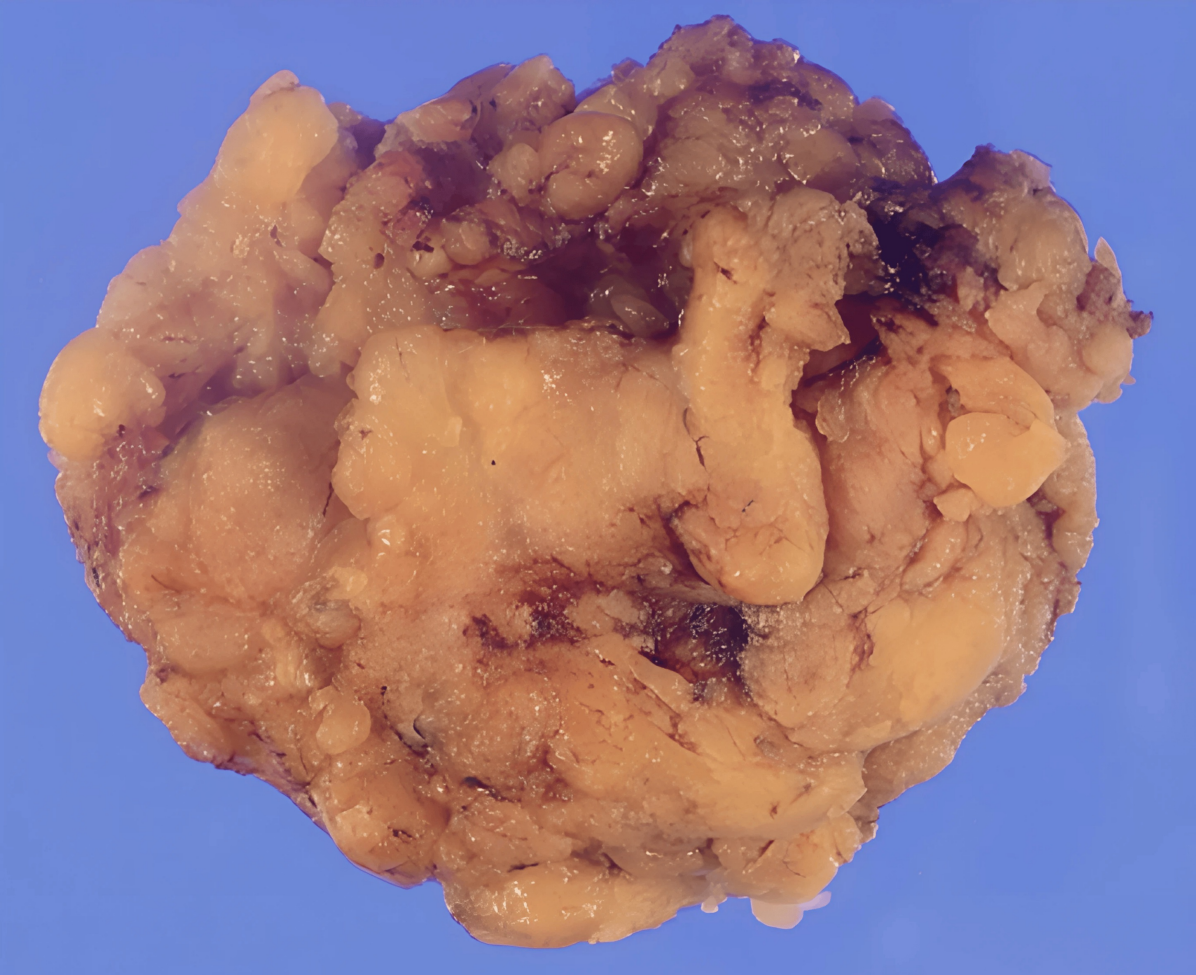
Initial surgical specimen Resection specimen from posterior cervical lipectomy demonstrating characteristic lobulated, yellow-tan appearance of benign adipose tissue (weight: 185 grams).

Following multidisciplinary consultation including anesthesiology assessment, posterior cervical lipectomy was performed under general anesthesia. Given the limited extent of disease at initial presentation, standard endotracheal intubation was performed without difficulty. A midline incision allowed for resection of adipose tissue down to the deep cervical fascia, with the tumor boundary being relatively clear from surrounding subcutaneous tissue. The resected specimen weighed 185 grams, displaying the characteristic yellow-tan color and lobulated texture of benign adipose tissue (Figure 2).

**Figure 3:**
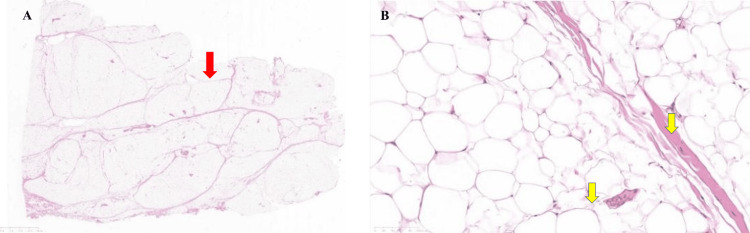
Histopathological evaluation (A) Low-power view (H&E, ×40) showing mature adipose tissue with preserved lobular architecture. (B) High-power view (H&E, ×100) demonstrating mature adipocytes without cellular atypia, consistent with BSL.

Histopathological analysis confirmed lobulated mature adipocytes without atypia and poor capsular formation, consistent with BSL (Figure 3A, 3B).

Postoperative recovery was uncomplicated with drain removal on postoperative day 5. Functional and cosmetic improvement was observed initially. The patient received counseling regarding alcohol cessation and was referred to addiction management and prevention services; however, adherence to abstinence recommendations could not be confirmed. Despite warnings about recurrence risk, subcutaneous fat gradually reaccumulated. The patient declined further surgery and was lost to follow-up for three years and six months postoperatively, until he re-presented with significant disease progression.

**Figure 4:**
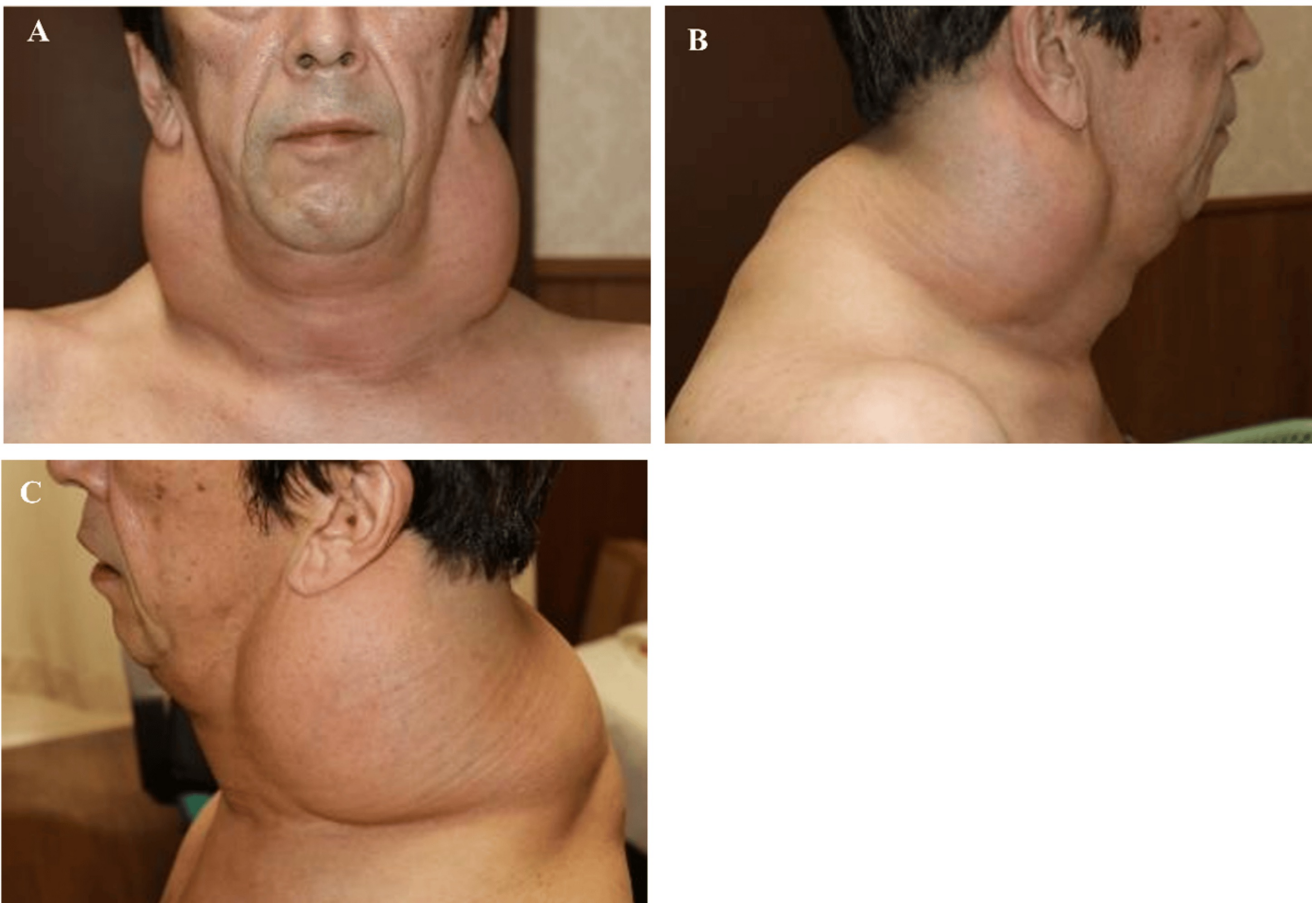
Clinical presentation at recurrence Clinical photographs at five years and five months postoperatively demonstrating marked bilateral cervical and upper back adipose proliferation: (A) anterior view, (B) right lateral view, and (C) left lateral-posterior view.

Five years and five months after the initial operation, the patient returned with marked progression of cervical and upper back swelling, new-onset dysphagia to solid foods, and worsening nocturnal respiratory distress requiring elevated head positioning during sleep (Figure 4A-4C). Physical examination revealed a firm, circumferential cervical mass with limited neck extension. Although formal sleep studies were not performed, the clinical history was highly suggestive of obstructive sleep apnea.

**Figure 5:**
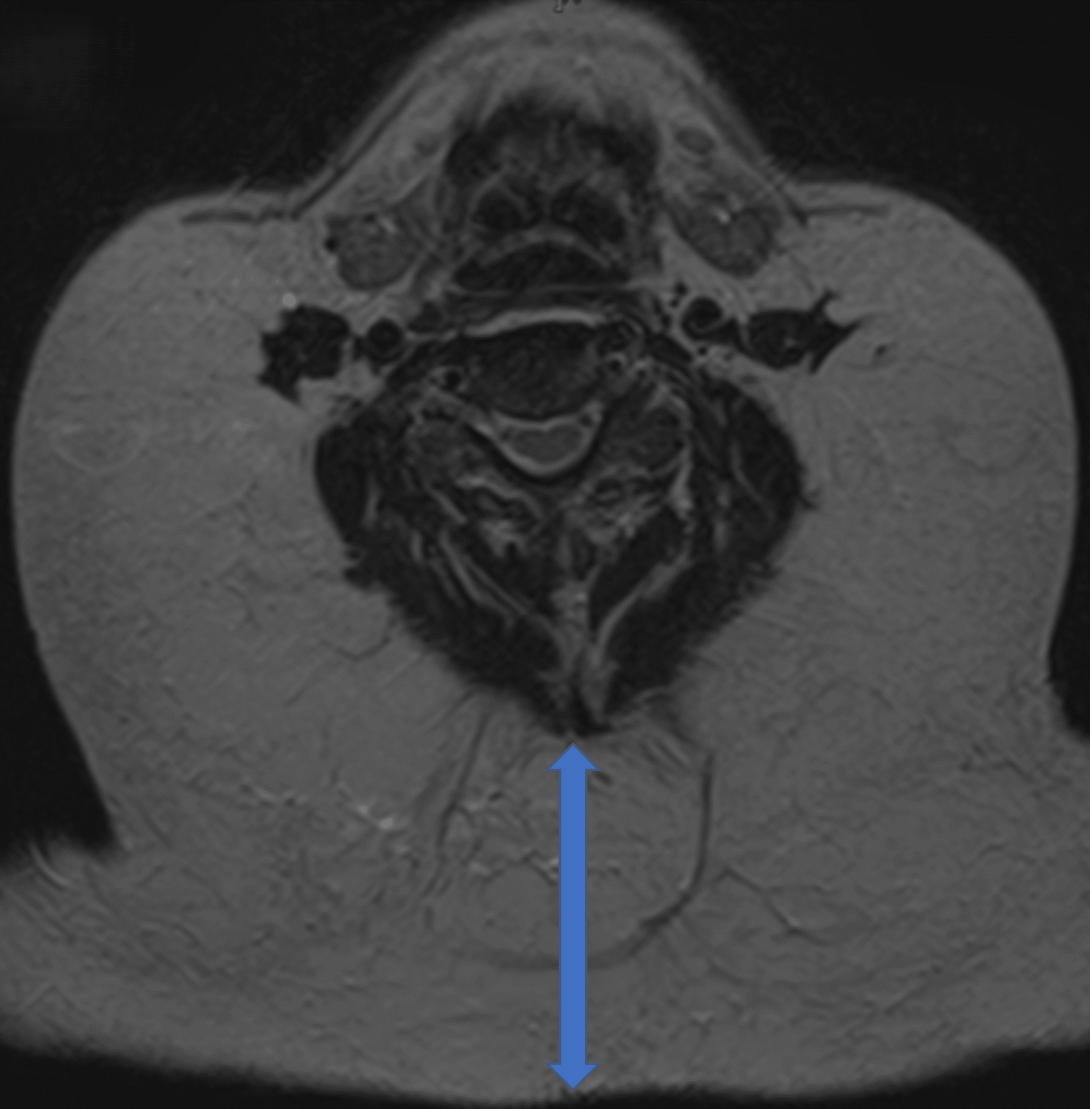
MRI imaging at recurrence T1-weighted axial image showing circumferential cervical adipose proliferation measuring 70 mm at the C4/5 vertebral level with mild airway compression.

MRI showed circumferential cervical adipose proliferation measuring approximately 70 mm in maximum anteroposterior diameter at the C4/5 level, representing a greater than three-fold increase from initial presentation (Figure 5). The mass effect resulted in mild compression of the airway and esophagus.

**Figure 6:**
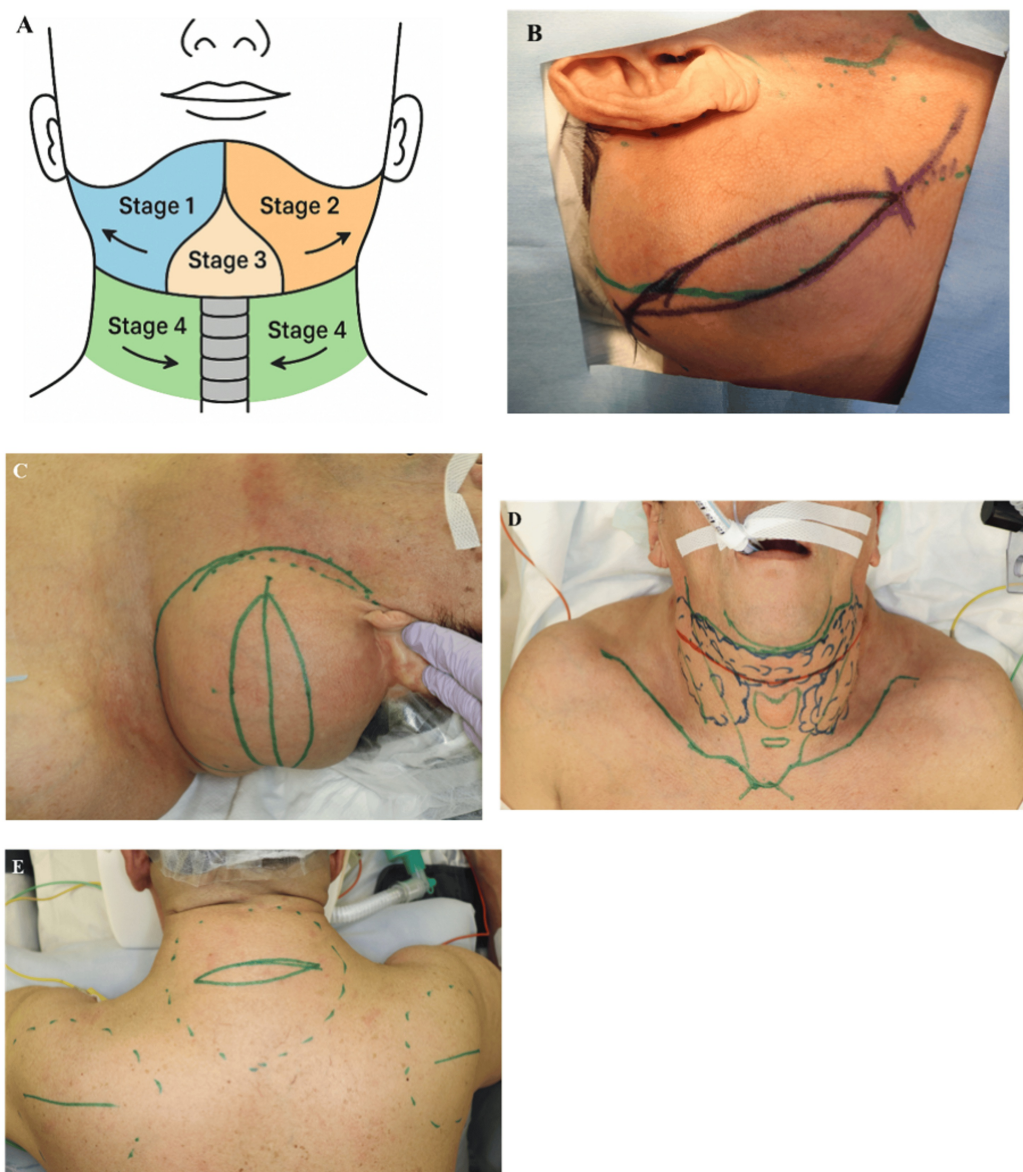
Systematic quadrant-based surgical planning approach (A) Schematic illustration of the planned four-stage surgical approach, divided by anatomical quadrants with annotated dissection depths. (B–E) Preoperative clinical photographs with detailed incision markings for each respective stage, illustrating the planned surgical boundaries and incision design strategy: (B) Stage 1 — right lateral quadrant, (C) Stage 2 — left lateral quadrant, (D) Stage 3 — central (submental) quadrant, and (E) Stage 4 — posterior cervical and scapular quadrants (preoperative view immediately before final surgical procedure, demonstrating extensive posterior adipose accumulation requiring fascial-level dissection). Original illustration created by the authors using Adobe Illustrator.

Given the extensive circumferential disease and the significant risk of airway compromise, a quadrant-based, four-stage open resection protocol was devised to mitigate perioperative risk (Figure 6A). Figures 6B-6E show preoperative skin markings with planned incision lines for each respective quadrant.

The surgical staging proceeded as follows with six-to-eight-week intervals between procedures. Stage 1 addressed the right lateral compartment in March 2023, Stage 2 the left lateral compartment in May 2023, Stage 3 the anterior submental region in August 2023, and Stage 4 the posterior cervical and scapular region in February 2024.

Intraoperative findings revealed variable adipose tissue depth distribution across anatomical regions: lateral compartments (Stages 1 and 2) were primarily superficial subcutaneous, while anterior and posterior regions (Stages 3 and 4) demonstrated deeper involvement requiring fascial-level dissection for complete excision.

Although specific documentation of intubation technique was not available in the medical records, the anesthesia team prepared for potential difficult airway management at each stage, with fiberoptic bronchoscope and emergency tracheostomy equipment readily available. Post-extubation monitoring in the intensive care unit for 24 hours was implemented after each procedure.

All procedures employed incisions along relaxed skin tension lines with careful preservation of key neurovascular structures, including the external jugular vein, spinal accessory nerve, and marginal mandibular nerve. En bloc adipose excision extended to the deep cervical fascia with preservation of superficial fascial planes to maintain cutaneous sensory innervation. Meticulous hemostasis and closed-suction drainage were employed at each stage. The total weight of excised tissue across all four stages was approximately 2,100 grams.

**Figure 7:**
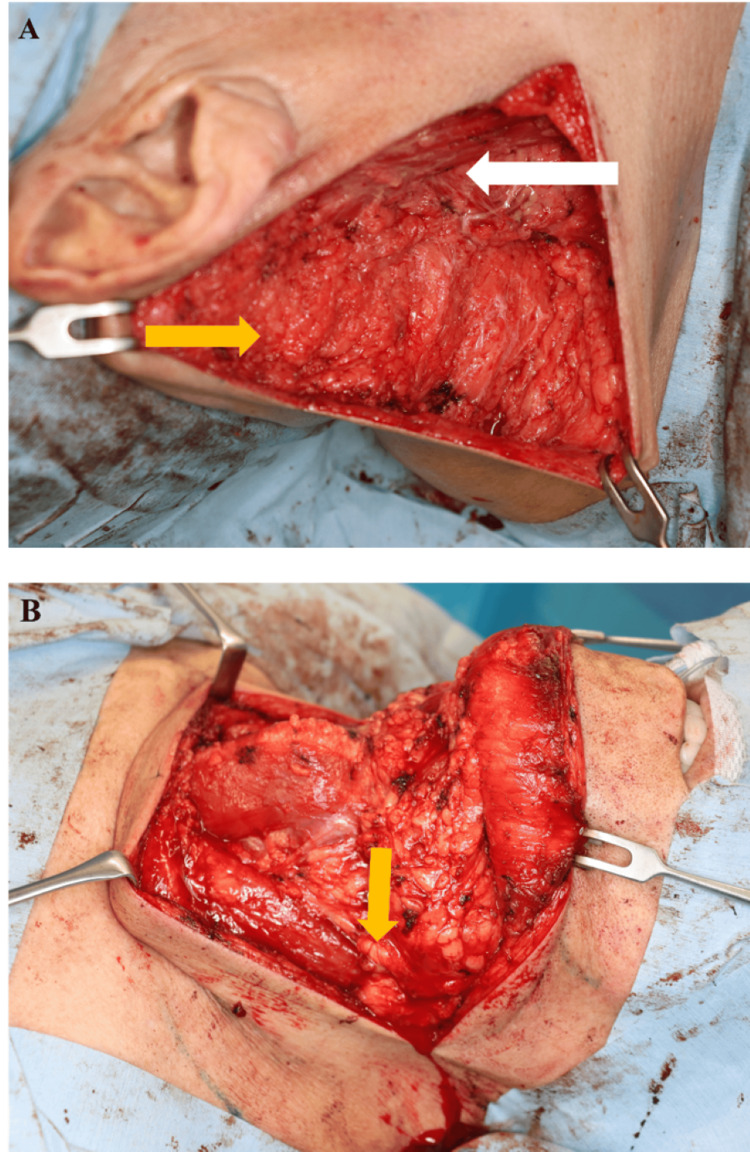
Intraoperative views of Stages 1 and 3 of quadrant-based resection (A) Intraoperative dissection of the right lateral compartment (Stage 1), revealing lobulated adipose proliferation (Yellow arrow) superficial to the sternocleidomastoid muscle (White arrow). (B) Intraoperative dissection of the central submental region (Stage 3), exposing subplatysmal adipose tissue extending toward the midline (Yellow arrow).

**Figure 8:**
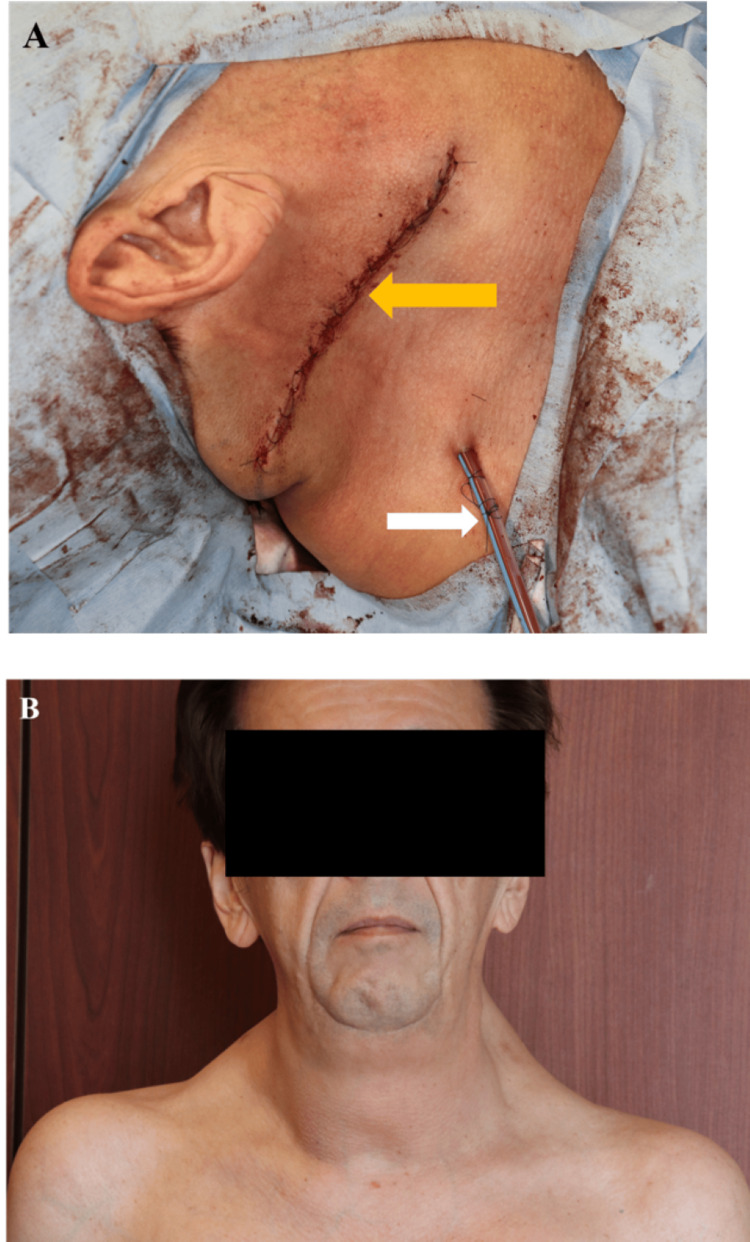
Postoperative outcomes of Stages 1 and 2 of quadrant-based resection (A) Immediate postoperative view after Stage 1 (right lateral quadrant) closure, demonstrating precise skin approximation (Yellow arrow) and subcutaneous drain placement (White arrow). (B) Early postoperative appearance following Stage 2 (left lateral quadrant), showing improved cervical contour and bilateral symmetry.

To illustrate the systematic surgical approach and progressive aesthetic outcomes achieved with our staged technique, representative surgical documentation is presented. Figure 7 and Figure 8 present detailed intraoperative and immediate postoperative outcomes. Figure 7 demonstrates critical technical elements including incision placement along relaxed skin tension lines, identification of anatomical boundaries such as the sternocleidomastoid muscle borders, systematic tissue dissection through clear planes, and preservation of major neurovascular structures including the internal carotid artery and jugular veins during Stages 1 and 3. Figure 8 presents immediate postoperative outcomes, showing lateral neck wound closure with drain placement (Figure 8A) and anterior view demonstrating overall neck contour improvement and residual submental fullness following the initial surgical stages (Figure 8B).

**Figure 9:**
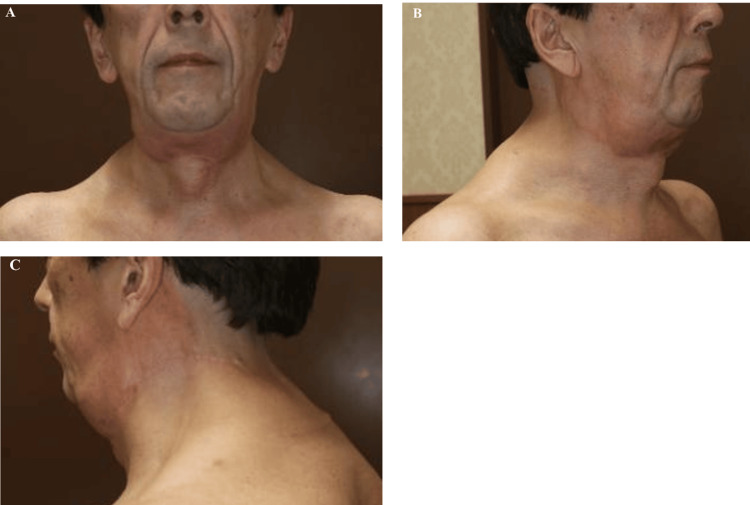
Postoperative clinical appearance at 16-month follow-up Clinical photographs demonstrating significant improvement in cervical contour with residual submental laxity: (A) anterior view, (B) right lateral view, and (C) left posterior oblique view.

**Figure 10:**
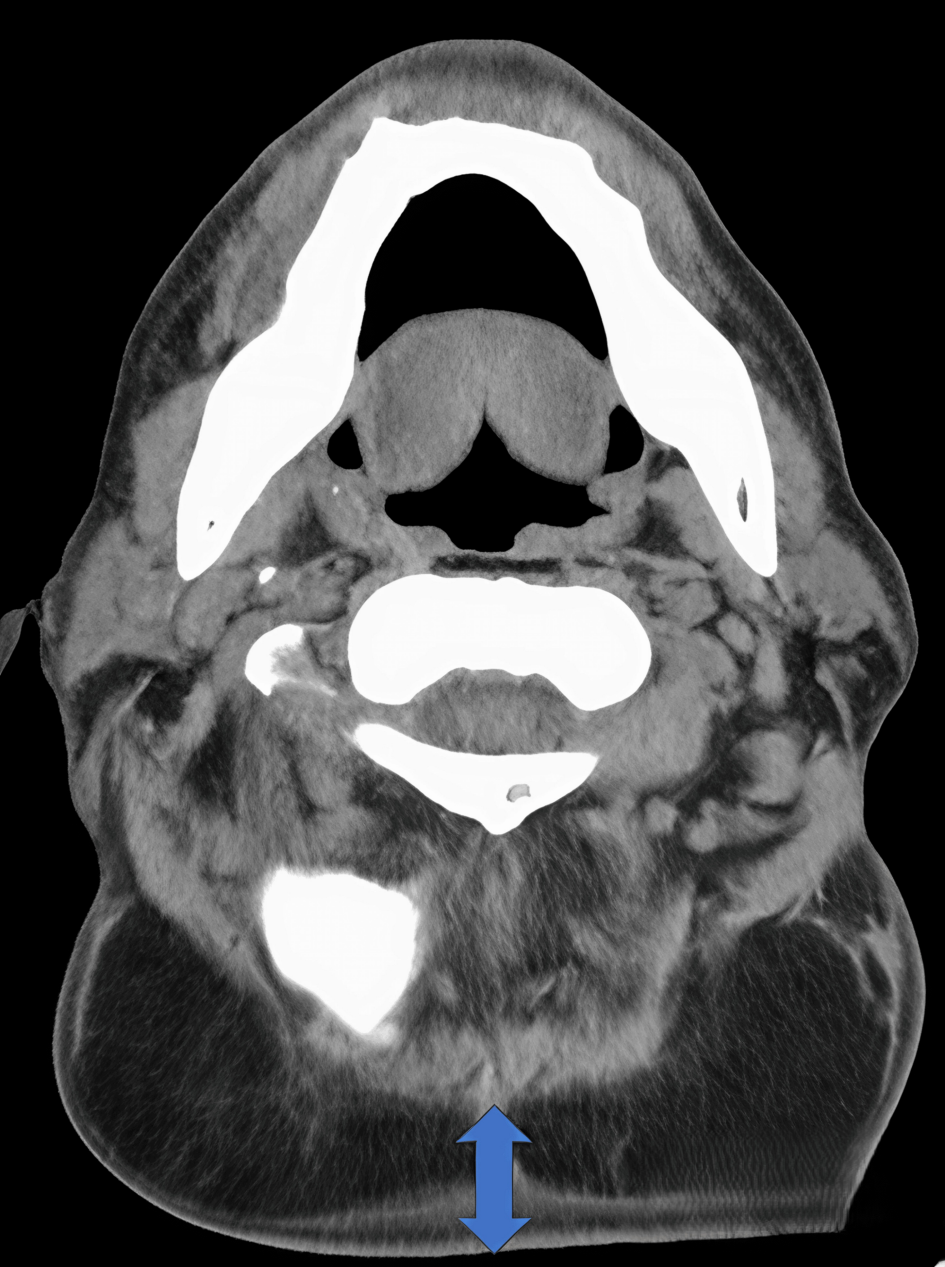
Comparative CT imaging Axial CT at the C4/5 level illustrating a reduction in cervical adipose thickness from 70 mm preoperatively to 48 mm postoperatively.

Histopathological examination of all resected specimens confirmed consistent findings of mature adipocytes without atypia, maintaining the diagnosis of BSL. At 16-month follow-up, CT demonstrated a reduction in cervical adipose thickness to 48 mm at the C4/5 level (Figures 9A-9C, 10). The patient reported complete resolution of dysphagia and marked improvement in sleep quality, though formal polysomnography was not performed to quantify the improvement.

Follow-up laboratory testing showed persistently elevated AST (76 U/L) and γ-GTP (134 U/L), raising concern for continued alcohol consumption. Mild renal function decline was also observed, with an estimated glomerular filtration rate (eGFR) of 84 mL/min/1.73 m^2^, possibly attributable to chronic alcohol exposure or age-related decline.

**Figure 11:**
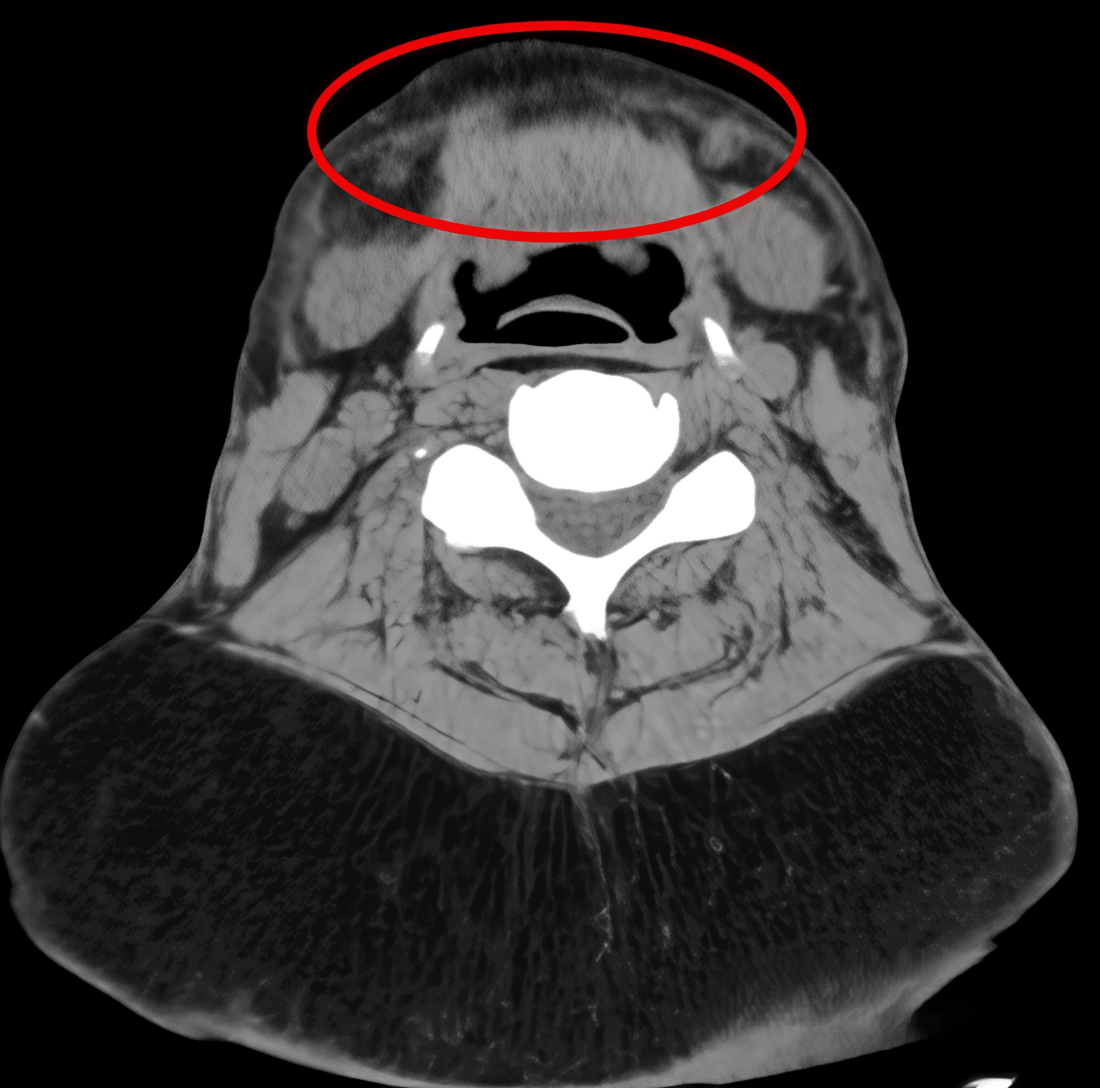
Postoperative CT imaging demonstrating platysmal anatomy Axial CT at the submental level showing midline platysmal diastasis (circled), contributing to residual soft-tissue laxity and neck contour irregularity.

**Figure 12:**
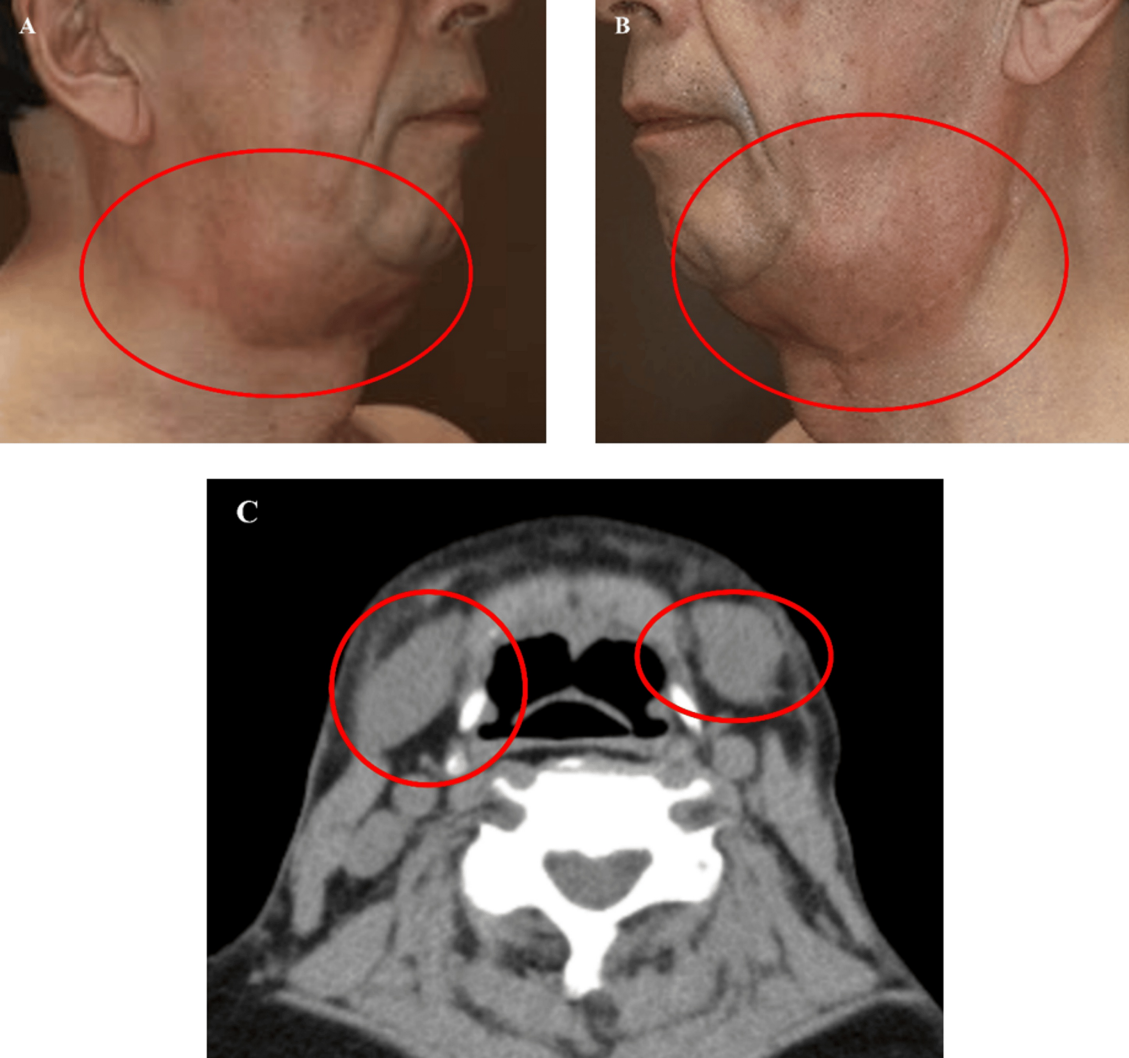
Submandibular gland imaging Clinical and radiological evaluation demonstrating bilateral submandibular gland enlargement contributing to residual submental fullness. (A, B) Preoperative clinical photographs showing gland prominence on the left and right sides, respectively. (C) Axial CT scan highlighting bilateral submandibular gland hypertrophy (circled in red).

Despite marked functional recovery, the patient expressed concern regarding residual aesthetic deformities, including submental laxity and transverse soft-tissue redundancy. Imaging evaluation revealed midline platysmal diastasis (Figure 11) and bilateral submandibular gland prominence (Figure 12A-12C) as contributing anatomical factors.

**Figure 13:**
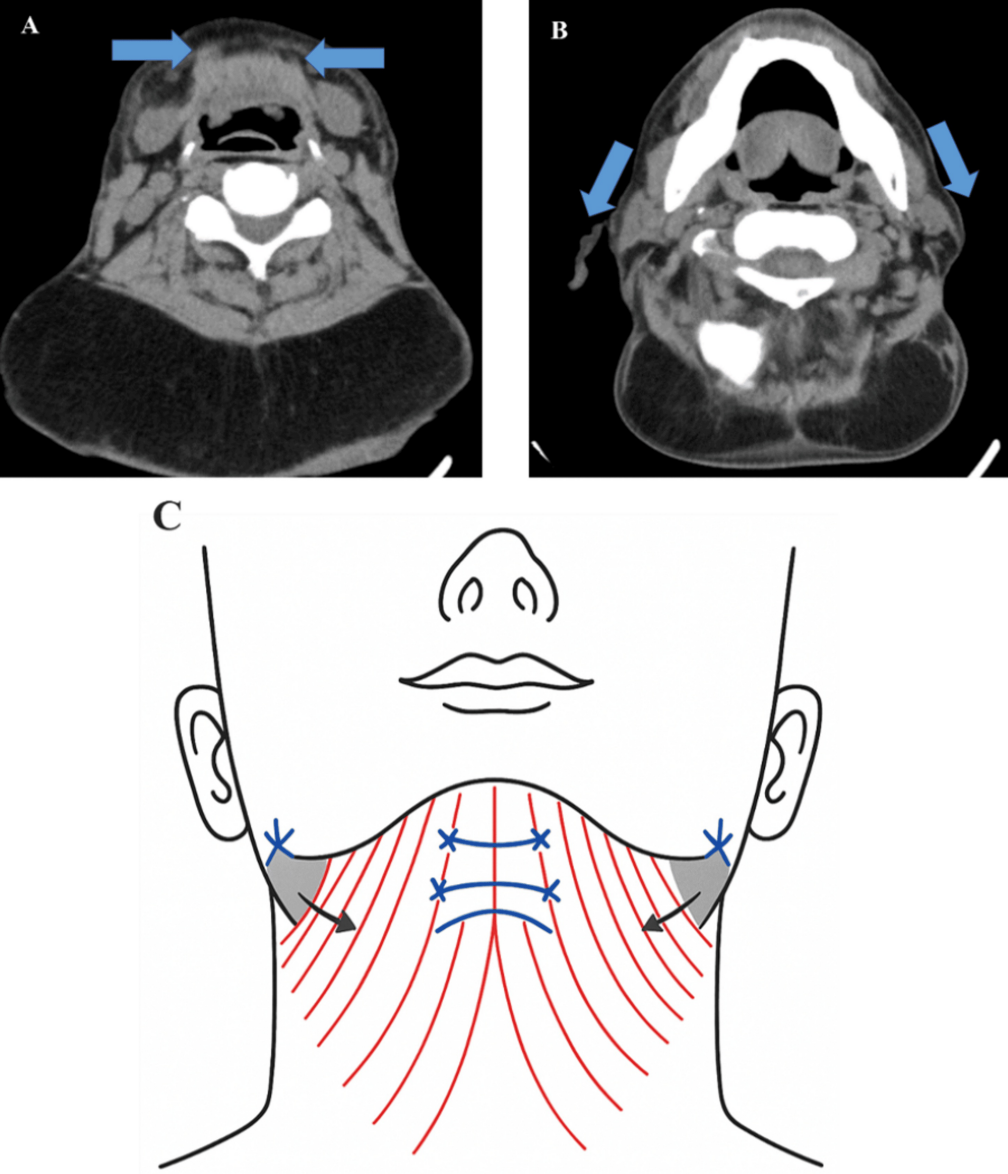
Proposed secondary surgical strategy Radiological and schematic representations of the planned corrective procedures targeting residual deformities: (A) Axial CT image showing midline platysmal banding, targeted for plication. (B) Axial CT image highlighting soft-tissue descent in the lateral neck, indicating areas for superolateral fascial suspension. (C) Schematic illustration of the comprehensive surgical plan, including midline platysmal plication, fascial suspension vectors, and partial submandibular gland reduction. Original illustration created by the authors using Adobe Illustrator.

**Figure 14:**
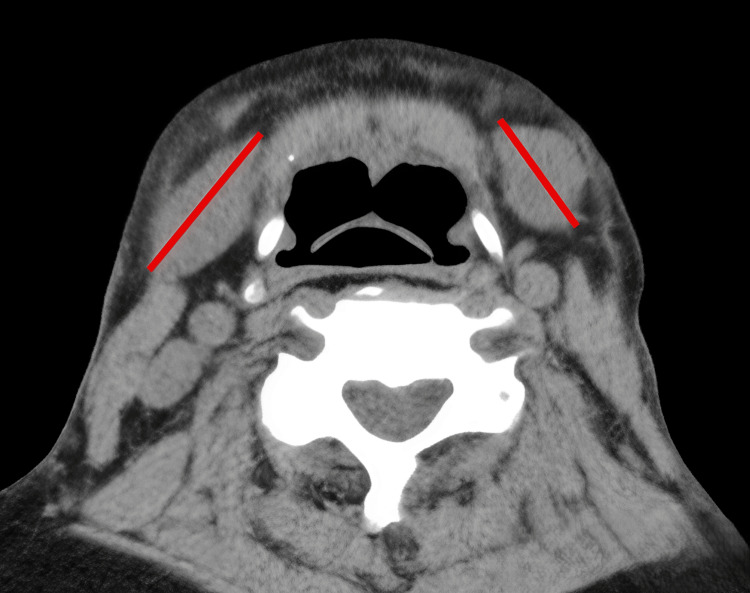
Submandibular gland reduction Axial CT image showing the proposed zone of partial submandibular gland reduction (incision line shown in red), intended to improve cervicomandibular definition and reduce bulk beneath the jawline.

A secondary surgical strategy was proposed, targeting the residual aesthetic deformities. The plan included midline platysmal plication, superolateral fascial suspension, and partial submandibular gland reduction, guided by both radiological assessment and intraoperative feasibility (Figure 13A-13C, Figure 14). However, given the persistently elevated γ-GTP suggesting ongoing alcohol consumption, these elective aesthetic procedures were deferred pending verified long-term alcohol cessation, due to potential implications for wound healing and recurrence risk.

## Discussion

Circumferential cervical BSL is an exceptionally rare clinical variant characterized by adipose tissue proliferation that completely encircles the trachea and esophagus, posing significant risks for airway obstruction and functional impairment [13,14,17-19]. The proximity of this adipose mass to vital neurovascular and airway structures complicates clinical management, particularly surgical intervention. The dramatic progression in our case from 21 mm to 70 mm of adipose thickness over five years underscores the potentially life-threatening nature of this condition when left untreated.

Although the underlying mechanisms of BSL have not been fully elucidated, strong associations with metabolic disorders, particularly chronic alcoholism, have been well documented [7,8]. Alcohol-induced suppression of β-adrenergic receptor activity and mitochondrial dysfunction contributes to abnormal proliferation of brown adipose tissue, predominantly localized to the cervicothoracic region [8,9,15]. Our patient's history of prolonged heavy alcohol consumption of 180 g of ethanol daily, persistently elevated γ-GTP levels ranging from 134 to 274 U/L during follow-up, and disease recurrence following initial surgery underscore the critical role of alcohol abstinence in disease control. The correlation between continued alcohol exposure and disease progression emphasizes the need for integrated addiction medicine services in managing BSL patients.

Surgical management remains the mainstay of treatment when functional impairment or significant cosmetic deformity occurs. Open lipectomy is considered the gold standard due to its capacity for en bloc excision with effective hemostasis. However, extensive circumferential disease presents unique challenges including difficult airway management, risk of bilateral recurrent laryngeal nerve injury, and potential for life-threatening postoperative edema. Our case required removal of approximately 2,100 grams of adipose tissue, highlighting the magnitude of surgical intervention needed in advanced cases.

The critical importance of airway management in circumferential BSL cannot be overstated. Despite the absence of formal documentation of our intubation approach, the preparation for difficult airway scenarios with fiberoptic bronchoscopy availability and 24-hour intensive care monitoring post-extubation proved essential. Future cases would benefit from standardized protocols including preoperative Mallampati classification, neck mobility assessment, and consideration of awake fiberoptic intubation as the primary approach.

A comparative analysis of previously reported cases of circumferential cervical BSL is summarized in Table 2. Prior reports predominantly describe single-stage or two-stage resections, often necessitating tracheostomy. In contrast, our quadrant-based, four-stage approach offers several clinical advantages. First, it reduces the risk of airway compromise by limiting surgical trauma and postoperative edema in each procedure. Second, it avoids tracheostomy through careful airway management and staged tissue reduction. Third, it enhances surgical precision and safety through sequential dissection of anatomically defined cervical compartments.

**Table 2:** Comparative Analysis of Documented Cases of Circumferential Cervical BSL BSL: Benign symmetric lipomatosis

Author (Year)	Country	Age/Sex	Clinical Presentation	Surgical Approach	Staging	Airway Management	Recurrence
Frąk et al. (2023) [16]	Poland	42/M	Neck swelling, limited movement	Open lipectomy	Two-stage	General anesthesia	No
Yan et al. (2022) [17]	China	65/M	Neck and upper back masses, mobility limitations	Open lipectomy	Single-stage	Standard intubation	No
Kitahara et al. (2022) [13]	Japan	64/M	Airway stenosis, obstructive sleep apnea	Open lipectomy, tracheostomy	Single-stage	Tracheostomy	No
Wanke and Yongjing (2021) [14]	China	51/M	Dysphagia, obstructive sleep apnea	Open anterior lipectomy, liposuction	Two-stage	General anesthesia	No
Present case (2025)	Japan	58/M	Dysphagia, nocturnal respiratory distress	Quadrant-based open lipectomy	Four-stage	General anesthesia	No (16 months)

The staging intervals allowed complete resolution of postoperative edema between procedures, though some centers may consider extending intervals for enhanced safety. This strategy achieved significant tissue reduction without recurrence at follow-up, accompanied by patient-reported functional improvements.

Aesthetic challenges persisted following mass resection, especially submental fullness and soft tissue ptosis. Imaging confirmed midline platysmal diastasis and submandibular gland prominence as contributing factors. A secondary corrective plan - including platysmal plication, fascial suspension, and partial gland reduction - was proposed, but deferred due to biochemical evidence of alcohol relapse.

These residual deformities resulted from anatomical changes following massive tissue resection. While therapeutic options range from non-invasive energy-based treatments to surgical correction - including platysmal plication and gland reduction [20] - we deferred these procedures given concerns about ongoing alcohol consumption. This decision highlights the ethical considerations in performing elective aesthetic procedures in patients with questionable adherence to medical recommendations.

Histopathological examination confirmed the benign nature of adipose proliferation without cellular atypia, effectively excluding malignant transformation such as liposarcoma. The characteristic lobulated architecture and mature adipocyte morphology of BSL were consistently observed across all surgical specimens [18].

The absence of formal polysomnography represents a significant limitation in our functional outcome assessment. Despite extensive counseling, the patient declined sleep studies due to personal and logistical concerns. While clinical indicators strongly suggested obstructive sleep apnea and objective improvements were documented (cervical thickness reduction from 70 mm to 48 mm, resolution of dysphagia), quantification of respiratory improvement remains unverified. Future protocols should mandate polysomnography as standard care, with early sleep medicine consultation and alternative assessment tools when formal studies are declined.

The absence of additional objective functional assessments - including fiberoptic endoscopic evaluation of swallowing and pulmonary function tests - also limits our ability to quantify the severity of preoperative impairment and postoperative improvement. Additionally, the lack of formal alcohol biomarker monitoring makes it difficult to confirm abstinence compliance, particularly given persistently elevated γ-GTP levels.

The relatively short follow-up period is insufficient to definitively assess long-term recurrence risk, particularly given reported recurrence rates of 30-60% in BSL patients who resume alcohol consumption. Furthermore, our single-case design inherently limits generalizability, though this limitation is unavoidable given the extreme rarity of circumferential cervical BSL.

Based on our experience, we recommend the following for managing similar cases: preoperative assessment should include formal polysomnography, swallowing studies, and detailed airway evaluation; objective alcohol monitoring using specific biomarkers should be implemented throughout treatment; imaging protocols should employ volumetric analysis rather than single-dimension measurements; surgical staging intervals of 12 weeks or more may provide additional safety margins; comprehensive documentation should be maintained; and a minimum three-year follow-up period is recommended.

The cumulative anesthetic exposure from multiple procedures necessitates careful patient selection and thorough cardiac evaluation, particularly in patients with comorbidities. Integration with addiction medicine services should be initiated at diagnosis rather than after surgical intervention, as sustained abstinence remains the cornerstone of preventing disease recurrence.

## Conclusions

Our quadrant-based, four-stage resection protocol successfully managed circumferential cervical BSL presenting with 70 mm of adipose tissue, causing dysphagia and respiratory distress. This approach avoided tracheostomy, preserved critical neurovascular structures, and achieved functional improvement with tissue reduction to 48 mm at 16-month follow-up. However, persistently elevated γ-GTP levels underscore that surgical success requires sustained alcohol abstinence to prevent recurrence. Future cases would benefit from objective functional assessments, alcohol biomarker monitoring, and more than 12-week staging intervals. This framework demonstrates that meticulous multidisciplinary planning, rather than surgical technique alone, determines outcomes in this rare but potentially life-threatening condition.
